# Engineered Promoter-Switched Viruses Reveal the Role of Poxvirus Maturation Protein A26 as a Negative Regulator of Viral Spread

**DOI:** 10.1128/JVI.01012-21

**Published:** 2021-09-09

**Authors:** Joe Holley, Rebecca P. Sumner, Sian Lant, Paolo Ribeca, David Ulaeto, Carlos Maluquer de Motes

**Affiliations:** a Department of Microbial Sciences, School of Biosciences and Medicine, University of Surrey, Guildford, United Kingdom; b Biomathematics and Statistics Scotland, The James Hutton Institute, Edinburgh, United Kingdom; c CBR Division, DSTL Porton, Salisbury, United Kingdom; d The Pirbright Institute, Woking, United Kingdom; University of Illinois at Urbana Champaign

**Keywords:** morphogenesis, poxvirus, vaccinia virus, virus transmission

## Abstract

Vaccinia virus produces two types of virions known as single-membraned intracellular mature virus (MV) and double-membraned extracellular enveloped virus (EV). EV production peaks earlier when initial MVs are further wrapped and secreted to spread infection within the host. However, late during infection, MVs accumulate intracellularly and become important for host-to-host transmission. The process that regulates this switch remains elusive and is thought to be influenced by host factors. Here, we examined the hypothesis that EV and MV production are regulated by the virus through expression of F13 and the MV-specific protein A26. By switching the promoters and altering the expression kinetics of F13 and A26, we demonstrate that A26 expression downregulates EV production and plaque size, thus limiting viral spread. This process correlates with A26 association with the MV surface protein A27 and exclusion of F13, thus reducing EV titers. Thus, MV maturation is controlled by the abundance of the viral A26 protein, independently of other factors, and is rate limiting for EV production. The A26 gene is conserved within vertebrate poxviruses but is strikingly lost in poxviruses known to be transmitted exclusively by biting arthropods. A26-mediated virus maturation thus has the appearance to be an ancient evolutionary adaptation to enhance transmission of poxviruses that has subsequently been lost from vector-adapted species, for which it may serve as a genetic signature. The existence of virus-regulated mechanisms to produce virions adapted to fulfill different functions represents a novel level of complexity in mammalian viruses with major impacts on evolution, adaptation, and transmission.

**IMPORTANCE** Chordopoxviruses are mammalian viruses that uniquely produce a first type of virion adapted to spread within the host and a second type that enhances transmission between hosts, which can take place by multiple ways, including direct contact, respiratory droplets, oral/fecal routes, or via vectors. Both virion types are important to balance intrahost dissemination and interhost transmission, so virus maturation pathways must be tightly controlled. Here, we provide evidence that the abundance and kinetics of expression of the viral protein A26 regulates this process by preventing formation of the first form and shifting maturation toward the second form. A26 is expressed late after the initial wave of progeny virions is produced, so sufficient viral dissemination is ensured, and A26 provides virions with enhanced environmental stability. Conservation of A26 in all vertebrate poxviruses, but not in those transmitted exclusively via biting arthropods, reveals the importance of A26-controlled virus maturation for transmission routes involving environmental exposure.

## INTRODUCTION

Members of the *Poxviridae* family are double-stranded DNA (dsDNA) viruses with a wide host range from insects (grouped in the subfamily *Entomopoxvirinae*) to vertebrates, including mammals, birds, and reptiles (grouped in the subfamily *Chordopoxvirinae*). The *Chordopoxvirinae* subfamily encompasses multiple genera, including *Orthopoxvirus* (OPXV), *Centapoxvirus*, *Yatapoxvirus*, *Leporipoxvirus*, *Capripoxvirus*, *Suipoxvirus*, *Cervidpoxvirus*, *Parapoxvirus*, *Molluscipoxvirus*, *Avipoxvirus*, and *Crocodylidpoxvirus*, plus a growing number of unclassified species (1). The biology and transmission modes of these vertebrate poxviruses are remarkably varied and can occur through direct contact, respiratory droplets, oral/fecal routes, or biting arthropods (2). The genus OPXV includes the infamous causative agent of human smallpox, variola virus (VARV), and the virus used for its eradication, vaccinia virus (VACV), as well as several other species, some of which can infect humans zoonotically, such as monkeypox virus (MPXV) or cowpox virus (CPXV) (3, 4). In addition, humans are also infected by molluscum contagiosum virus (MCV), which causes benign infections that self-resolve over time.

Due to its role in smallpox eradication and its enormous potential as a vaccine vector, VACV has become the best studied and prototypic poxvirus, although the natural host and the origins of VACV remain unclear. VACV undergoes a very complex replication and morphogenic cycle that occurs exclusively in the cell cytosol and involves multiple maturation stages. The final outcome of this process is the formation of two antigenically distinct virion forms known as extracellular enveloped virus (EV) and intracellular mature virus (MV) (5). EVs are released during infection and mediate virus spread to neighboring cells, becoming essential for viral dissemination within an infected individual. On the contrary, MVs are retained inside the cell in much higher numbers and are only released at the end of the infection cycle when cells lyse. In some species, an accumulation of MV inside a dense proteinaceous cytosolic structure can be seen. This structure, known as the A-type inclusion (ATI) (originally referred to as Downie bodies [6] or Marchal bodies [7]), is thought to provide stability and enhanced protection to mediate virus transmission between individuals through the environment. Therefore, while EVs are important for dissemination, MVs are important for transmission.

The process that mediates the formation of EV and MV upon entry and establishment of cytosolic viral factories is complex and has been studied intensely (reviewed elsewhere [8–10]). Initially, newly assembled virus cores acquire one membrane marked with the virus protein A27 (8). A27 forms trimers that attach to the virus membrane via protein A17 (11–14) and is essential in allowing transport of virions to the *trans*-Golgi network (15–17). Here, virions are further wrapped to eventually become EV, double-membraned virions that are released rapidly for dissemination in an individual and in cell culture (18–20). The formation of EV is chiefly controlled by protein F13 (21, 22), a nonglycosylated viral protein that associates with Golgi membranes through posttranslational palmitoylation (23–25). F13 is essential for further wrapping of single-membraned virions (26, 27), and its absence leads to a dramatic reduction of viral spread and plaque size (21, 22). EVs exiting the plasma membrane remain cell associated or are pushed for longer dissemination via actin tails (20, 28, 29). While EVs are released rapidly, single-membraned MVs progressively accrue inside the cell until lysis and spread only very slowly between cells. These MVs are generally considered wrapping-competent virions that for unknown reasons do not progress to EVs. Interestingly, a subset of these MVs acquires proteins that are not present in any EV membrane, namely, A26 and A25, suggesting an alternative route of maturation (30). A25 is the truncated homolog of the occlusion factor responsible for ATI formation in some OPXV, such as CPXV or ectromelia virus (ECTV) (31–33), whereas A26 is the molecular bridge that mediates occlusion of virions into ATI (34). A26 binds MV via C-terminal disulfide bonds with the surface protein A27, whereas the N terminus binds the ATI (35–38). In OPXV lacking ATIs, such as VACV or VARV, the ATI protein is truncated so its C terminus that is required for oligomerization and ATI formation is lost. However, the N terminus (referred to as A25) is still expressed and retains its ability to associate with the virion via A26 (36). A26 is therefore conserved and binds MV in OPXV proficient and deficient for ATIs.

The presence of unique virus proteins on, and distinct functions for, the two morphotypes suggested to us that passive production from a leftover is an unlikely replication strategy, and virions containing A26 might be an actively regulated virus form. F13 and A26 are late genes that are transcribed after virus DNA replication. However, A26 production is detected much later than F13 (30) and correlates with the progressive accumulation of MV inside the cell. In addition, the interaction between A26 and A27 suggested that A26 could block the functions of A27, which are required for the formation of EV. This possibility has, however, been dismissed so far because absence of A26 did not enhance EV production (35, 38, 39). Here, we hypothesized that A26 positively regulates the formation and accumulation of MV and that its deletion results in a larger pool of undifferentiated nascent virus, but not in an increase of EV, which would require positive regulation by F13. To test this hypothesis, we engineered recombinant virus with altered kinetics of expression for A26 and F13. We found that viruses expressing A26 and F13 simultaneously had fewer EV and reduced spread, and this correlated with enhanced A26/A27 complex formation. Therefore, during infection, viral progeny acquire A27 and can become EV (in a process that depends on and is coeval with F13 expression) or mature into A26-positive MV as A26 expression initiates. The two processes coexist during viral infection thanks to the regulation of expression of the key differentiation factors F13 and A26. Intriguingly, gene *A26L* is absent in *Chordopoxvirus* genera known to be transmitted by arthropods, suggesting that these groups have evolved alternative life cycles perhaps as an adaptation to the obligate use of vectors.

## RESULTS

### Generation of promoter-swapped viruses for F13 and A26.

**FIG 1:**
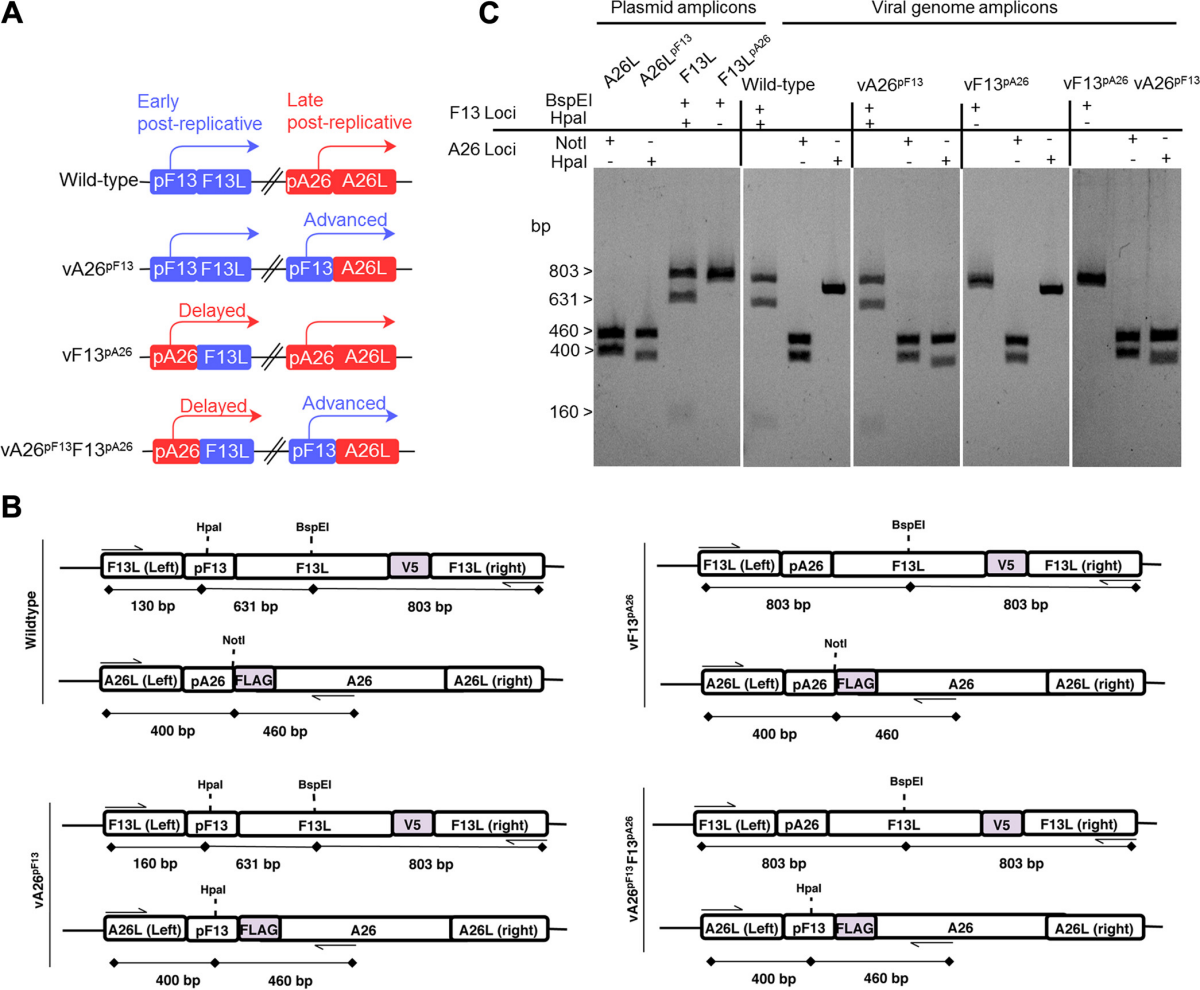
Generation of promoter-swapped viruses with altered kinetics of expression for F13 and A26. (A) Recombinant viruses in which the promoter of the F13 gene (blue) and the promoter of the A26 gene (red) were swapped. (B) Genomic organization of the A26 and F13 loci in each of the engineered viruses. The genetic identity of the promoters introduced in the promoter-swapped recombinant viruses was verified by restriction enzyme digestion of PCR amplicons produced with oligonucleotides flanking the promoters (indicated by arrows). Size of the amplicons and the expected digested fragments are indicated. (C) Digestion of viral genome PCR amplicons with the indicated restriction enzymes. All viruses yielded the expected restriction pattern according to their promoter configuration and as observed from digestion of plasmid amplicons obtained from transfer vectors.

Our hypothesis was that A26 interacts with MV and prevents their F13-mediated maturation into EV, so A26-mediated and F13-mediated maturation are mutually exclusive. This is currently difficult to explore because the expression of the two proteins initiates and peaks at different times postinfection, with A26 being expressed substantially later than F13 (30). Our hypothesis would, however, predict that synchronizing A26 expression with that of F13 would negatively impact EV production and virus spread, resulting in smaller plaques on cell monolayers, which is controlled by EV (19, 21, 22). To test this, we engineered viruses in which the promoters of A26 and F13 were switched (Fig. 1A). We generated a virus in which the promoter of A26 was replaced for by the promoter of F13 (vA26^pF13^), so A26 expression was now advanced and synchronized with F13 expression. We also generated a virus in which the promoter of F13 was replaced for by the promoter of A26 (vF13^pA26^), so F13 expression was now delayed and synchronized with that of A26. Finally, a virus was created in which both promoters were swapped (vA26^pF13^F13^pA26^). To facilitate relative detection and quantitation of both A26 and F13, promoter-switched viruses expressed FLAG-tagged A26 and V5-tagged F13, so a control virus expressing tagged genes with promoters in wild-type configuration (WT-analog) was also generated and used throughout the study. The insertion of these transcriptional elements and tags created sites for unique restriction enzymes as detailed in Fig. 1B, which were exploited for genetic analysis of the engineered viruses. Specific oligonucleotides were designed to amplify the *F13L* and *A26L* loci, and purified PCR amplicons were subsequently digested with the corresponding restriction enzymes (BspEI and HpaI for the *F13L* locus and NotI and HpaI for the *A26L* locus). Viral genome amplicons from all viruses presented the expected restriction pattern compared with the restriction obtained with the plasmids used for recombination (Fig. 1C).

### Kinetics of F13 and A26 mRNA and protein expression from promoter-swapped viruses.

**FIG 2:**
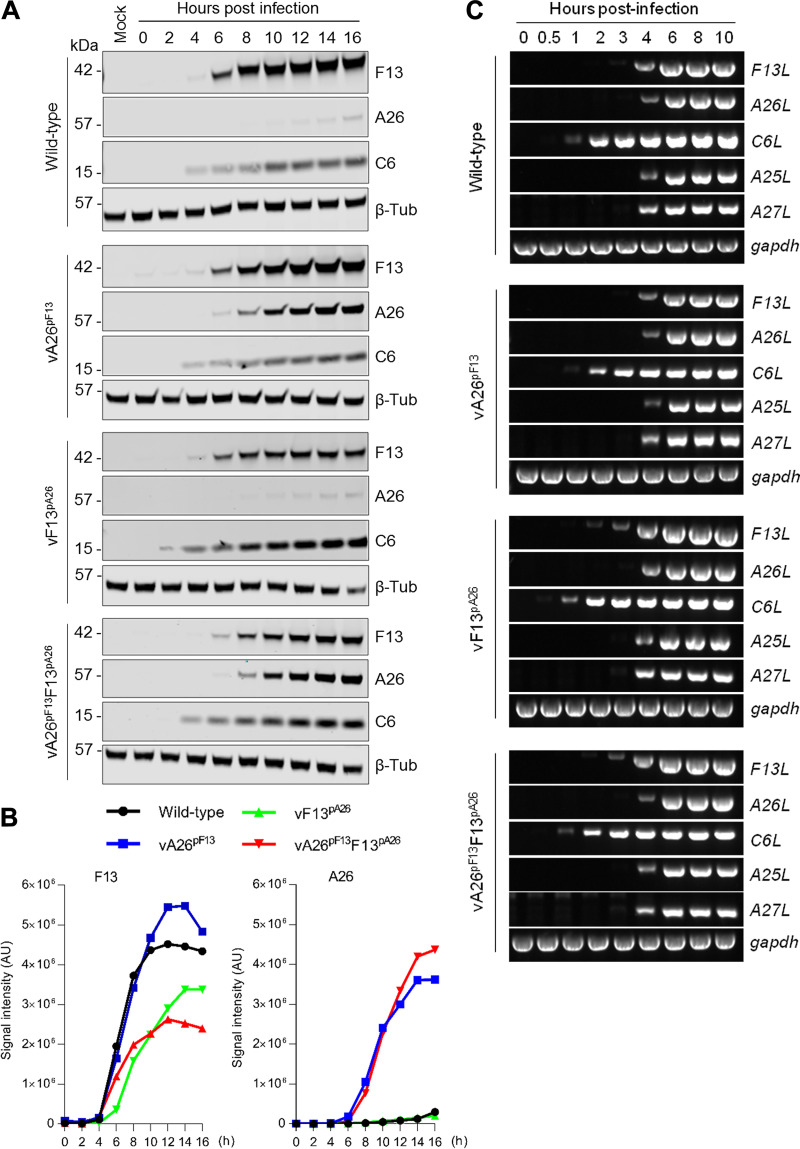
Viruses expressing A26 under the control of the F13 natural promoter expressed A26 much earlier and at similar kinetics to F13. (A) Cells were infected with 5 PFU/cell of the indicated viruses. At the indicated hour postinfection, cells were lysed and extracts analyzed by quantitative infrared immunoblotting for the expression of A26 and F13 as well as the control viral protein C6 and β-tubulin (β-Tub). (B) Quantitative analysis of A26 and F13 expression over time. Data from (A). Signal intensity (arbitrary units [AU]) recorded for A26 and F13 in each of the indicated virus kinetics was normalized to C6 and plotted over time. Expression of F13 was slightly delayed in vF13^pA26^ viruses, whereas expression of A26 was advanced in vA26^pF13^ viruses. (C) RT-PCR for the indicated viral genes and *GAPDH* was performed on cDNA from cells infected with 2 PFU/cell of the indicated viruses for the indicated length of time. Amplicons were visualized on agarose gels using SYBR-Safe stain. In all cases, one out of two experiments with similar results is shown.

To determine whether the relative kinetics of F13 and A26 production had been altered, we performed time course expression analyses by immunoblotting infected cell extracts (Fig. 2A). In line with previous reports, wild-type virus produced detectable levels of F13 from 6 h, whereas A26 accumulated much later at around 16 h postinfection. vA26^pF13^ also expressed F13 from 6 h but expressed A26 significantly earlier than the WT-analog and at a similar time to F13 in agreement with the replacement of the natural A26 promoter for that of F13. In vF13^pA26^, a virus in which F13 expression was controlled by the A26 promoter, F13 was expressed only slightly later than F13 in the WT-analog, although the intensity of the bands was clearly reduced. This was unexpected and suggested that additional transcriptional mechanisms for the *F13L* gene in the F13 locus may exist. Finally, these observations were recapitulated in the double promoter-switched virus (vA26^pF13^F13^pA26^). The kinetics of expression of A26 and F13 in each virus were quantitated using quantitative infrared fluorescence imaging, and this confirmed the changes of expression introduced by each promoter alteration, including the slight delay and reduced intensity in F13 expression in vF13^pA26^ (Fig. 2B). Importantly, all viruses expressed the early viral protein C6 at 2 to 4 h, demonstrating that the modifications did not affect relative virion infectivity.

We also analyzed the expression of *F13L* and *A26L* from these viruses at the mRNA level. We detected the *F13L* mRNA at 4 h postinfection when F13 expression remained under the control of the F13 promoter, such as in wild-type virus infection (as previously reported [40–43]), and with vA26^pF13^ but also in the viruses where F13 expression was controlled by the A26 promoter (vF13^pA26^ and vA26^pF13^F13^pA26^) (Fig. 2C). Detection of the *A26L* mRNA also occurred at 4 h postinfection for all the engineered viruses (Fig. 2C). This indicated that the promoters of A26 and F13 drive mRNA production at the same time, in agreement with their postreplicative nature (40–43). Detection of mRNA from other postreplicative genes, such as *A27L* and *A25L*, also occurred at this time, in contrast with early genes such as *C6L*, which is expressed much earlier (Fig. 2C). These results indicated that the kinetics for F13 mRNA and protein production were similar even when the *F13L* gene was controlled by the A26L promoter, which can be explained by the fact that both F13 and A26 promoters drive expression at the same time or by read-through from upstream promoters as previously identified (41, 43). The kinetics for A26 mRNA and protein, however, differed ostensibly, and while A26 mRNA expressed from the *F13L* promoter produced detectable protein shortly after, it produced protein much later when expressed from its natural promoter. The discrepancy between *A26L* mRNA synthesis and A26 protein accumulation has already been shown by others and was proposed to be caused by A26 protein instability (42). Our results suggest that further regulatory mechanisms control the expression of A26.

### Advanced expression of A26 reduces viral spread.

**FIG 3:**
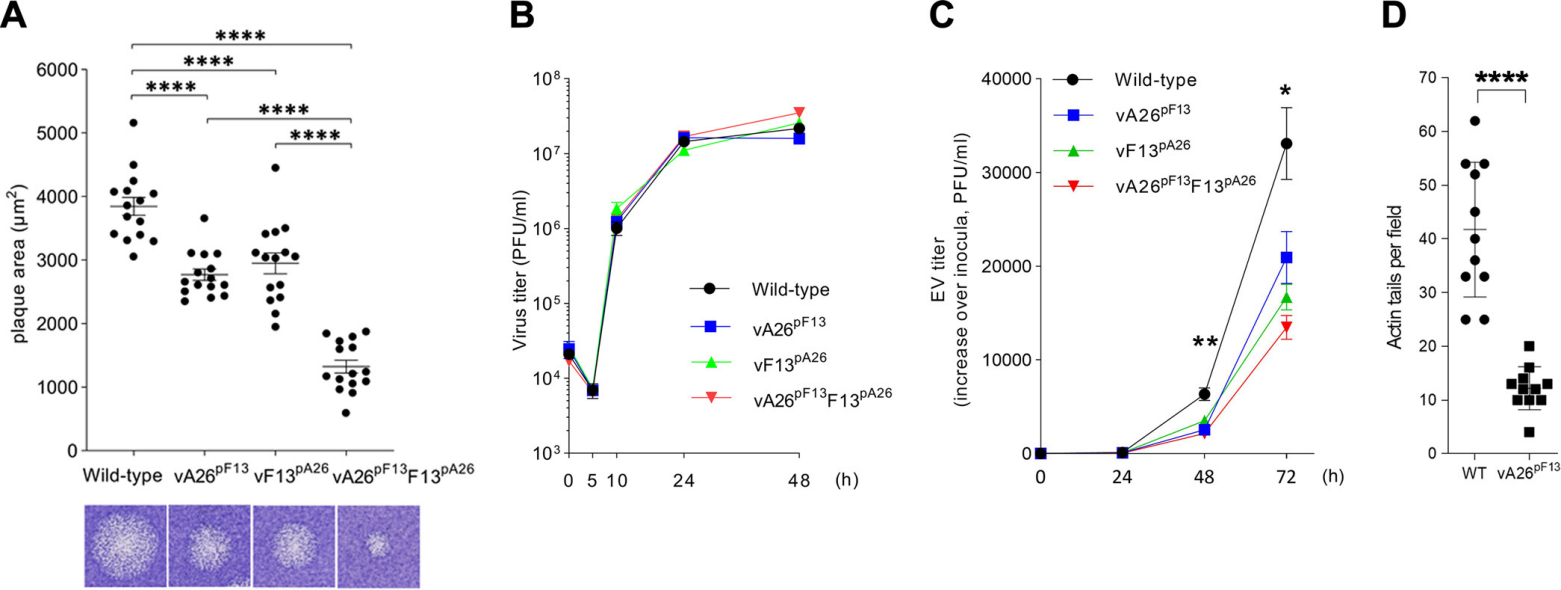
Advanced expression of A26 reduces plaque size, EV formation, and actin tail projections. (A) Analysis of viral spread by plaque assay. Cells were infected with the indicated viruses and incubated in semisolid overlay for 72 h. The area of viral plaques (*n* = 15) was measured and compared by Student’s *t* test. A representative plaque is shown underneath the graph. Significant differences in viral spread were observed in all promoter-swapped viruses compared to the wild-type virus (****, *P* < 0.0001). (B) One-step viral growth curves. Cells were infected with 5 PFU/cell of the indicated viruses in triplicate, and progeny virus was titrated at the indicated hours postinfection and plotted as mean and standard deviation (SD). No significant differences in growth were observed between the viruses. (C) Multistep growth curves. Cells were infected with 0.001 PFU/cell of the indicated viruses in triplicate, and EV in the medium was titrated at the indicated hours postinfection and plotted as mean and SD (fold increase over time). A significant difference was observed between vA26^pF13^ and wild-type virus using a Student’s *t* test (*, *P* < 0.05; **, *P* < 0.005). (D) The number of actin tails observed during wild-type (WT) and vA26^pF13^ infection was recorded and compared using Student’s *t* test (****, *P* < 0.0001). Advanced expression of A26 in vA26^pF13^ reduced the number of actin tails, a process known to be mediated by EV.

We then examined the ability of these viruses to spread. Monolayers of cells were infected with the different viruses, and viral spread was determined by the size of the plaques 3 days after infection. All promoter-switched viruses showed altered plaque size relative to WT-analog (Fig. 3A), indicating that the modifications had altered the kinetics of virus maturation. vF13^pA26^ had significantly reduced plaque size, likely reflecting the slight delay and reduced intensity of F13 expression (Fig. 2B). This confirmed the enormous impact that F13 levels have on EV formation and plaque size. Remarkably, vA26^pF13^, which expressed F13 as usual but A26 earlier, also displayed reduced plaque size. Consistent with this, the modifications introduced into vA26^pF13^F13^pA26^ had a cumulative effect, and this virus had the most profound effect on maturation, showing smaller plaques than WT-analog and both the single-switch viruses. To determine if plaque size reductions resulted from reduced overall virus production, we performed one-step growth curves, where cells were infected with 5 PFU/cell, and viral progeny was quantitated at different times postinfection. All viruses, including those where A26 expression had been advanced, gave similar numbers of total progeny (Fig. 3B). This demonstrated that the overall virus yield obtained from these viruses was unaffected and that the changes observed in plaque size were attributable to EV levels, which account for viral spread. To address this, we infected cells with 0.001 PFU/cell and quantitated the levels of EV in culture supernatants over 3 days by plaque assay. We observed reduced EV production in all engineered viruses, including vA26^pF13^, compared to wild-type virus over time, and this reached statistical significance at 48 h postinfection (Fig. 3C). In addition, we measured the number of actin projections that are produced when EV are propelled from the cell for dissemination (20, 29). We found these to be significantly lower in vA26^pF13^-infected cells (Fig. 3D), supporting the overall effect that advanced A26 expression inflicts on virus spread.

**FIG 4:**
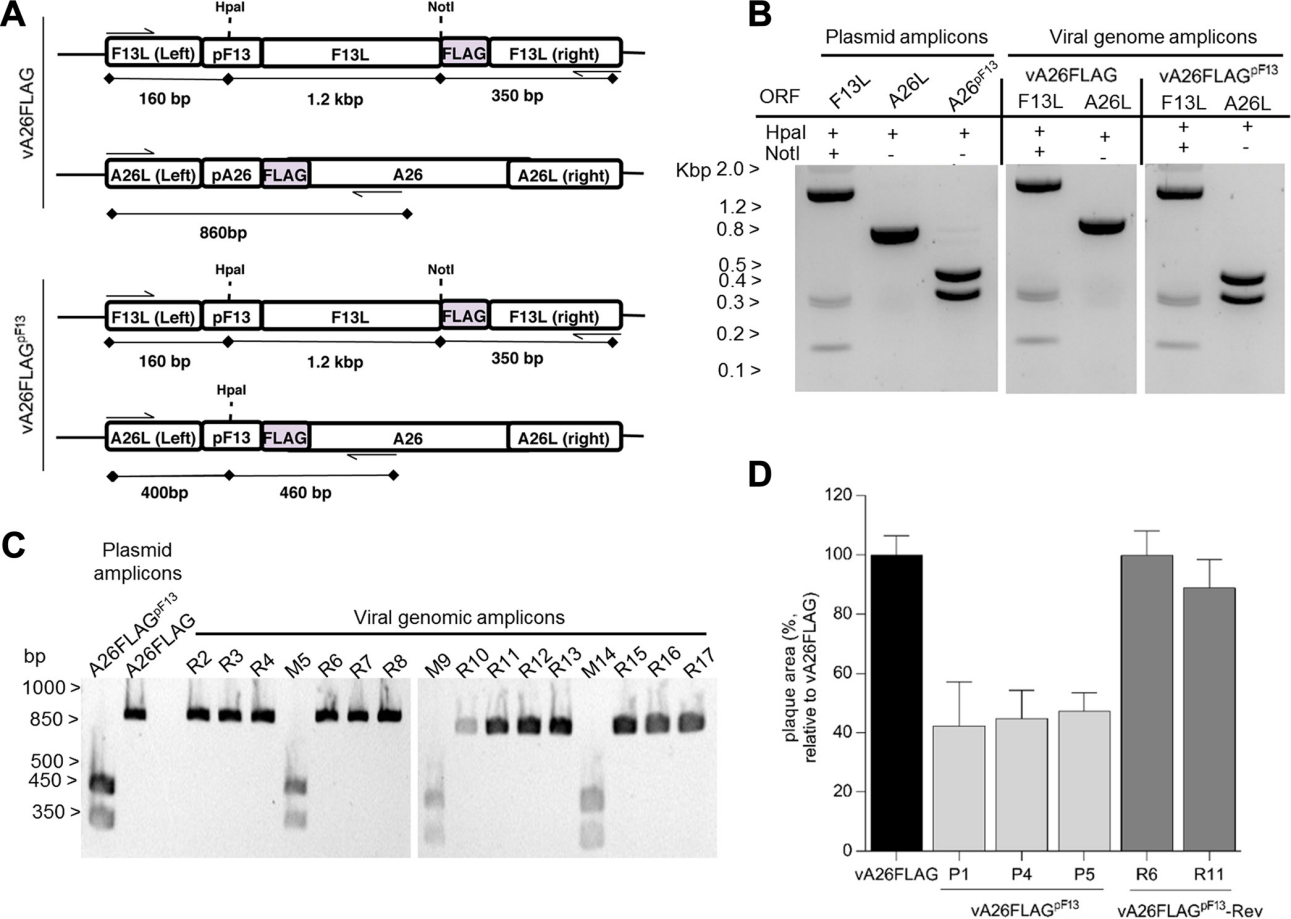
Reinstating A26 promoter into the A26 locus restores plaque size. (A) Genomic organization of the engineered recombinant viruses expressing FLAG-tagged A26 under the control of the A26 promoter (vA26FLAG) or the F13 promoter (vA26FLAG^pF13^). (B) The genetic identity of the promoters introduced in the recombinant viruses was verified by restriction enzyme digestion of PCR amplicons produced with oligonucleotides flanking the promoters (indicated by arrows). The sizes of the amplicons and the expected digested fragments are indicated. (C) Restriction enzyme digestion of viral genome PCR amplicons from a number of plaque-purified revertant viruses (labeled R) or viruses retaining the mutant genotype (labeled M) corresponding to vA26FLAG^pF13^. Amplicons from revertant viruses failed to digest (like the parental vA26FLAG) as a consequence of reinstating the natural A26 promoter. (D) Analysis of viral spread by plaque assay. Cells were infected with the original vA26FLAG, vA26FLAG^pF13^ (3 representative isolates), and vA26FLAG^pF13^-Rev (2 representative isolates) and incubated in semisolid overlay for 72 h. The area of viral plaques (*n* = 15) was measured and presented as a percentage of the size obtained for vA26FLAG. Plaque area for vA26FLAG^pF13^ viruses was greater than 50% smaller than original vA26FLAG, and this was restored in vA26FLAG^pF13^-Rev isolates.

To further validate these results, we generated a second set of viruses where F13 and A26 incorporated the same FLAG epitope so that relative protein levels could be assessed using the same antibody. Following a similar strategy as above, a virus was created in which expression of FLAG-tagged A26 was advanced by replacement of its natural promoter by that of the *F13L* gene (vA26FLAG^pF13^). A control virus expressing FLAG-tagged A26 under the control of its natural promoter (vA26FLAG) was also created (Fig. 4A). The genetic identity of these viruses was verified by restriction of each locus amplicon and was found to be the same as the control plasmids (Fig. 4B). We also created a revertant virus in which the natural promoter of the *A26L* gene was put back into its natural locus (vA26FLAG^pF13^-Rev). Genetic analysis of several plaque-purified revertant viruses yielded a restriction pattern similar to the original vA26FLAG (Fig. 4C). When virus spread was assessed, replacement of the A26 promoter with that of F13 in vA26FLAG^pF13^ advanced A26 expression (not shown) and caused plaque size reduction (Fig. 4D). Importantly, reinstating the A26 native promoter restored A26 expression kinetics and plaque size to that of the WT-analog, demonstrating that the virus phenotypes were caused by the intended A26 promoter alterations. Taken together, these results demonstrated that A26 had the capacity to limit EV formation and suppress virus spread.

### Advanced A26 complexes with A27 and prevents F13 incorporation into virions.

**FIG 5:**
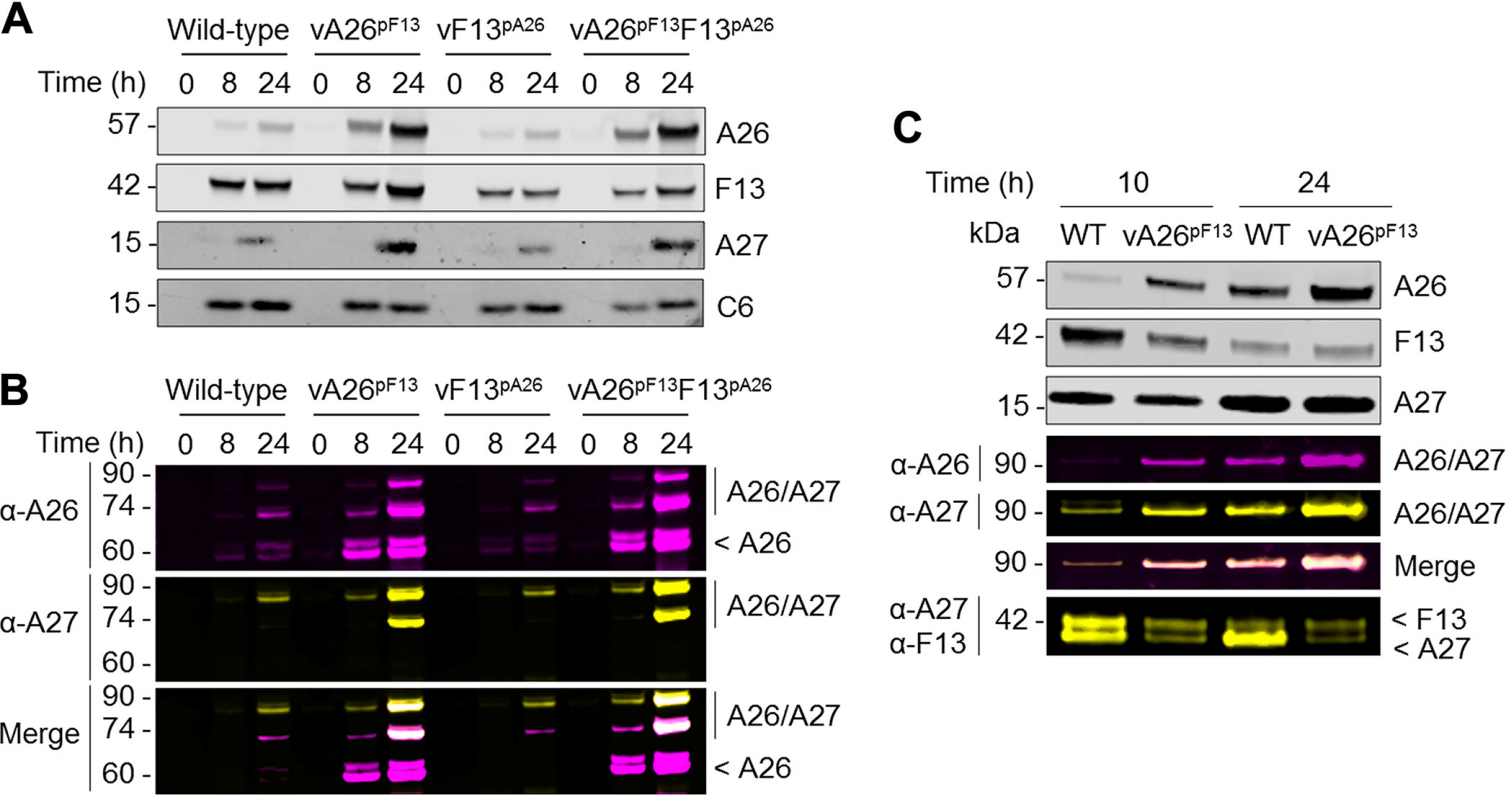
Advanced A26 complexes with A27 and excludes F13 incorporation. Cells were infected with the indicated viruses for the indicated length of time, and lysates were analyzed by SDS-PAGE under denaturing (A) and nondenaturing (B) conditions for the expression of the viral proteins F13 (V5), A26 (FLAG), and A27 as well as the early protein C6, used as a control. Molecular weights (kDa) are indicated on the left. In agreement with their promoter configuration, A26 expression was enhanced in vA26^pF13^ viruses, whereas F13 expression was slightly delayed in vF13^pA26^ viruses. Where A26 expression was advanced, it correlated with increased detection of A26-A27 complexes. (C) Cells were infected with WT-analog (WT) and vA26^pF13^ viruses for the indicated length of time, and progeny virus was purified and analyzed by SDS-PAGE under denaturing and nondenaturing conditions for the expression of the indicated proteins. Progeny virus from vA26^pF13^ infection incorporated significantly higher levels of A26 that correlated with a decrease in F13 detection.

Next, we investigated the mechanism underlying A26-mediated suppression of viral spread. The viral A27 trimer on the surface of MV mediates transport to the wrapping sites where F13 locates (15). Furthermore, A26 is anchored on the MV surface through direct interaction with A27 (35). So, A27 is associated with both routes of maturation. To study the effects of A26 on A27 with our set of promoter-switched viruses, cells were infected with each of our viruses for 8 or 24 h, and cell lysates were processed under reducing conditions (Fig. 5A) as well as nonreducing conditions to preserve the A26/A27 association (Fig. 5B) as previously reported (35). In agreement with promoter configurations, F13 kinetics were retarded, and levels were reduced in vF13^pA26^ and vA26^pF13^F13^pA26^ extracts, whereas A26 expression was advanced and increased in vA26^pF13^ and vA26^pF13^F13^pA26^ extracts. Levels of A27 were also slightly increased in vA26^pF13^ and vA26^pF13^F13^pA26^ infections and were likely stabilized by advanced A26 expression. Immunoblotting for A26 (Fig. 5B, purple) and A27 (Fig. 5B, yellow) was also performed on nonreduced samples. These blots revealed the expected 60-kDa band for A26 but also bands at 75 and 90 kDa corresponding to the size of complexes of A26 disulfide bonded with A27 dimers and trimers. These higher-molecular-weight bands were particularly prominent in vA26^pF13^ and vA26^pF13^F13^pA26^ extracts at 24 h postinfection and were positive for both A26 and A27 (Fig. 5B, merge panel). This demonstrated that advanced A26 retained the ability to associate with A27 and that vA26^pF13^ formed A26/A27 complexes indistinguishable from those of WT, but earlier.

Infected cell lysates contained a mixture of A26 and A27 proteins that were incorporated into virions or remained soluble in the cell. To discard the excess of soluble protein and further assess the impact of advanced A26 on virus maturation, we lysed infected cells with hypotonic buffer and subjected cell homogenates to a 36% sucrose cushion before immunoblotting under reducing and nonreducing conditions (Fig. 5C). At 10 h postinfection, WT-analog infection resulted in barely detectable A26 but significant levels of F13, indicating that a large proportion of virions undergo F13-mediated wrapping to become EV at this time point. As expected, at 24 h postinfection, the A26 signal was much more pronounced. Remarkably, vA26^pF13^ progeny virions contained a substantially larger amount of A26 than WT-analog. Concomitant with this elevated A26 incorporation, a decrease in F13 incorporation was observed, particularly at 10 h postinfection. Nonreducing blots demonstrated that incorporated A26 was in a 90-kDa complex with A27, which led to a decrease in noncomplexed trimeric A27 levels relative to WT-analog. Thus, A26 association with A27 correlates with an impairment in F13 recruitment. Collectively, these findings indicate that expression of A26 (advanced by virtue of the *F13L* promoter) correlates with its packaging and with the exclusion of F13, eventually impacting EV levels, plaque size, and virus spread.

### A26 is conserved in OPXV but is absent in clade II poxvirus.

**FIG 6:**
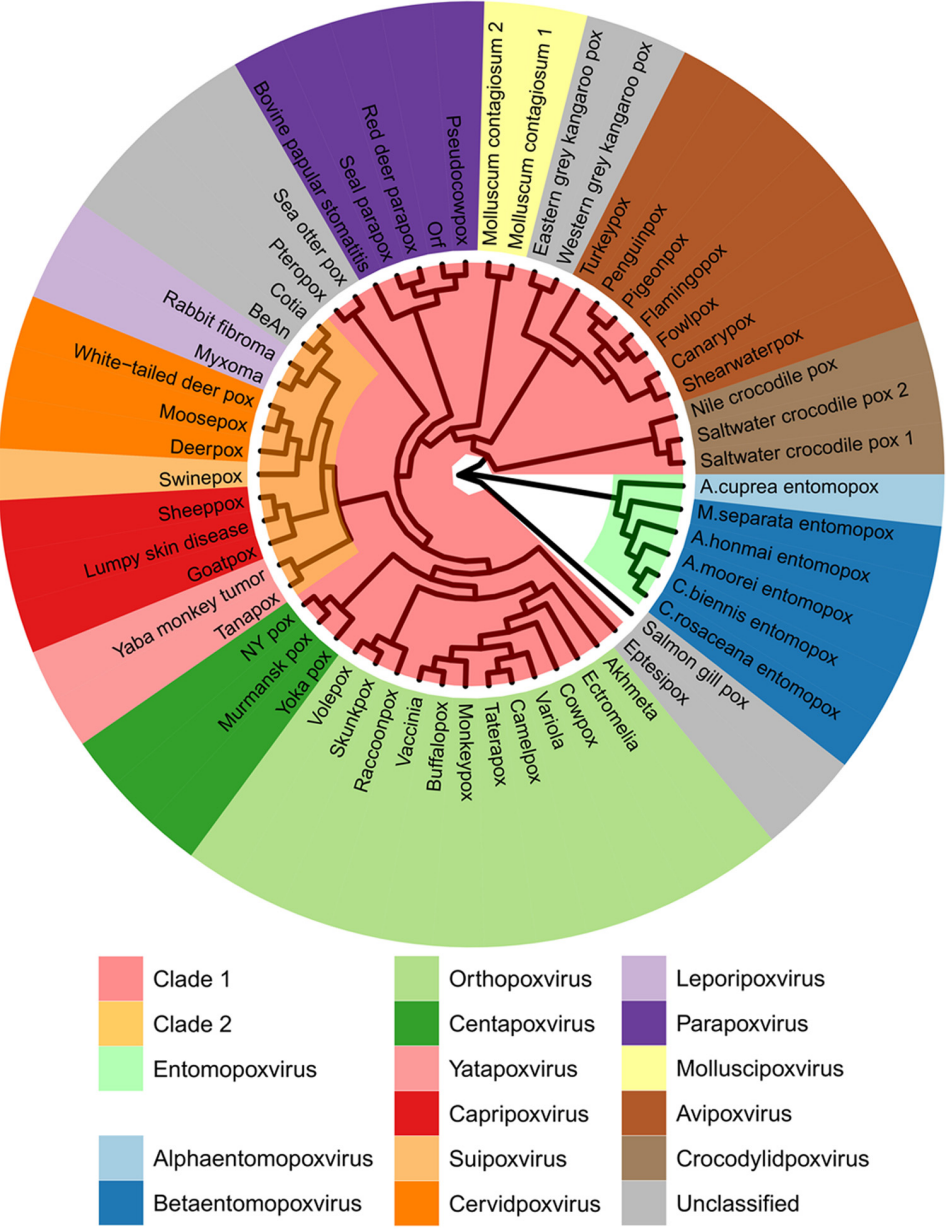
Phylogenetic relationship between poxvirus species indicates that A26 is not ancestral to the chordopoxviruses. Vinyl plot depicting the phylogenetic relationship between representative poxvirus species based on the orthologs of the E9 gene. The inner ring depicts a maximum likelihood tree based on the JTT model of amino acid evolution (see Table 1 for sequence accession numbers). Branch lengths are not to scale. Clade I chordopoxviruses are shown in pink, and clade II chordopoxviruses are in yellow. The outer ring depicts a list of representative virus species color-coded based on genera.

**FIG 7:**
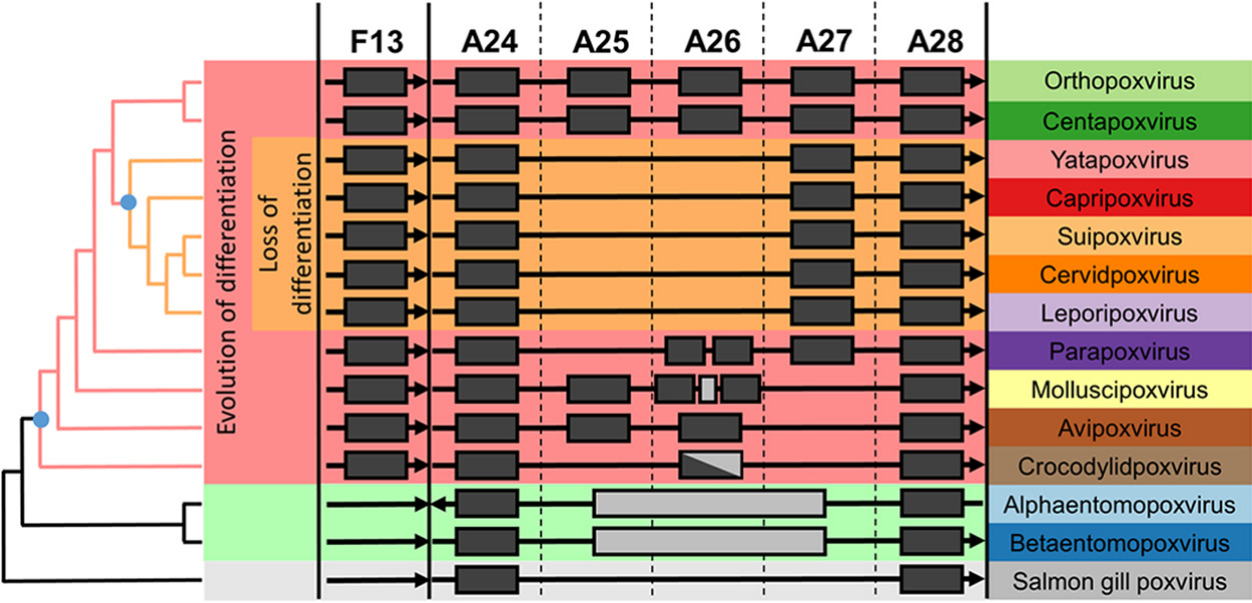
A26 is conserved in chordopoxviruses with the exception of arthropod-transmitted clade II chordopoxviruses. Occurrence of the indicated loci in established poxvirus genera. Genes with significant homology present in a genus are denoted by filled black rectangles; gray rectangles indicate extraneous nonhomologous sequence. The A24 and A28 genes immediately flank the virus differentiation locus in chordopoxviruses. The F13 EV-associated gene is separated from the differentiation locus by ∼100 kb in chordopoxviruses, depending on the genus. The A25/A26 differentiation system is present in virtually all known clade I chordopoxviruses (excluding crocodylidviruses) and absent in all known clade II chordopoxviruses. Parapoxviruses lack the A25 gene but have two nonidentical A26 homologs in tandem. Molluscipoxviruses have two A26 homologs and an intervening unrelated ORF. Molluscipoxviruses, avipoxviruses, and crocodylidviruses lack A27. Crocodylidviruses lack A25 and have a longer, weakly homologous ORF in lieu of A26. Entomopoxviruses lack the F13 gene as well as the A25/A26 differentiation locus, which is replaced by several ORFs showing no homology; Alphaentomopoxviruses have A24 and A28 swapped. Phylogenetic distances are not to scale, and virus genera are arranged according to phylogeny of the E9 DNA polymerase gene.

Given the importance of F13 and A26 as factors driving the maturation of poxvirus virions, we examined their presence and conservation across the family. Chordopoxviruses (vertebrate poxviruses) are divided into clades based on genome-wide gene conservation (44, 45). In agreement with previously reported phylogenies (44–48), phylogenetic analysis using the virus DNA polymerase and the major core proteins A3 and A10 confirmed that OPXVs belong to clade I, whereas clade II includes the *Yatapoxvirus*, *Capripoxvirus*, *Suipoxvirus*, *Cervidpoxvirus*, and *Leporipoxvirus* genera (Fig. 6 and data not shown). These analyses indicated that the clade II poxviruses are not ancestral to the chordopoxviruses. We were able to identify F13 homologs across all chordopoxviruses (Fig. 7), indicating the conserved function of this gene. Conversely, A26 was much more variable. A26 was conserved in OPXVs and centapoxviruses; however, it was completely absent in clade II chordopoxviruses (Fig. 7). In fact, clade II viruses lacked homologs of both genes *A25L/ATI* and *A26L*, which are closely related and are likely to have arisen as a duplication of an original single gene. Strikingly, these loci were completely missing in all clade II genera, with *A27L* typically starting closely after *A24R* termination or even overlapping by a few nucleotides. Homologs of the OPXV A26 were found in parapoxviruses, molluscipoxviruses, avipoxviruses, and even crocodylidpoxviruses, albeit with weaker homology. Consequently, it appears that the A25/A26 differentiation locus is ancestral to chordopoxviruses and was lost early in the radiation of clade II viruses. Chordopoxviruses generally transmit through direct contact, respiratory droplets, or oral/fecal routes, modes of transmission likely to benefit from the enhanced environmental stability provided by A25/A26. Interestingly, where it is known, clade II viruses are arthropod transmitted (49–56). Loss of the differentiation system in clade II viruses may thus be an adaptation to obligate transmission by biting arthropods in which infected cells may be the infectious vehicle, which would make A25/A26 functions superfluous. Although clade I avipoxviruses exhibit arthropod transmission, they are also transmitted by other routes (57, 58).

## DISCUSSION

Chordopoxviruses produce two types of mature infectious virions with one or two membranes. The biogenesis of these virions is regarded as a sequential process in which single-membraned MVs acquire a second membrane to become EVs or are retained in the cell as MVs. This process has traditionally been thought to be regulated by host factors, such as the availability of cellular membranes required for wrapping, with the initial MV becoming an infectious precursor of EV and the remaining MV progeny becoming the leftover cytosolic virus, which can make up 99% of total progeny in VACV (19). In this model, the single-membraned MV form is seen as a passive remnant virus that is unused and accrues when the cellular membranes required for the double-membraned form are exhausted. In some OPXVs, such as ECTV, CPXV, or raccoonpox virus, these accrued MVs become occluded into cytoplasmic ATI (6, 7, 31). Here, we show that the viral protein A26 skews virus maturation toward the single-membraned MV form and antagonizes maturation of the alternative double-membraned EV form. The ability of A26 to negatively regulate wrapping and EV formation seems at odds with previous literature reporting that A26 deletion viruses did not enhance EV titers (35, 38, 39). However, A26 does not kinetically regulate EV production, and its deletion may lead to the accumulation of infectious EV precursors requiring positive regulation by F13 to fully mature into EV. Our results demonstrate that if synchronized with F13, expression of A26 follows similar kinetics as that of F13, but it complexes with A27 earlier and prevents F13 incorporation, thus reducing EV formation and plaque size. Therefore, engineered promoter-synchronized viruses demonstrate the capacity of A26 to negatively regulate viral spread. The delayed kinetics in the expression of A26 compared to F13 during natural infection initially prevents the antagonistic effect of A26 and allows for sufficient virus dissemination via EV before maturation then switches to predominantly A26-positive MV. Thus, as previously reported (19, 30), EV production peaks in the earlier postreplicative stages and is followed by the accumulation of MV concomitant with A26 expression as the infection progresses in the cell. In ATI-positive viruses, this process leads to MV occlusion into ATI. However, the majority of OPXVs have a truncated ATI protein, A25, that does not aggregate into ATI and yet retain full-length A26, indicating that A26-mediated maturation is a biologically important function independent of ATI formation.

The whole process has similarities to the process of cellular differentiation observed in Metazoa in that an undifferentiated pluripotent entity (here, the single-membraned virus containing A27) has the ability to give rise to two antigenically and functionally distinct forms (here, the double-membraned EV and the single-membraned virus containing A26) in response to differentiation factors (here, viral proteins F13 and A26, respectively). Although similar analogies can be drawn to spore-forming bacteria or the latent and lytic phases of herpesviruses and papillomaviruses, fundamental differences exist in that bacterial sporulation and viral reactivation are sequential processes and do not involve the parallel production of different morphotypes from a single progenitor. OPXV virion maturation may therefore be the closest process to metazoan-like differentiation observed outside metazoan eukaryotes. The adaptive value of this bifurcated maturation pathway is hypothesized to be balancing intrahost dissemination and host-to-host transmission in cellular environments that may vary depending on cell type and whole host factors, such as health, nutritional status, or immune status. In addition, A26 is known to regulate viral entry through pH-dependent conformational changes that trigger membrane fusion at low pH (39, 59). The presence of A26 on MV may thus also represent a strategy to prevent premature virus-cell fusion within the parent infected cell and to facilitate entry into subsequent cells through the endocytic pathway.

The genomic loci between homologs of the VACV genes *A24R* and *A28L* exhibit remarkably high variability across the different poxvirus genera. The gene *A26L* is conserved across the OPXV, although it is mutated in certain VACV (e.g., Copenhagen) and CPXV (e.g., Brighton-Red) strains and has been reported to become inactivated in *in vitro* experimental settings (59, 60). While these mutations might be a reflection of passage and experimentation in cell culture where the benefit of A26-positive virions with enhanced environmental stability for transmission is negligible, we were surprised to observe that the entire A25/A26 loci were absent in all clade II poxviruses, which encompass five genera and several unclassified species. The A26 locus is ancestral to chordopoxviruses, and we found homologs of the OPXV A26 in other genera (excluding entomopoxviruses and the unclassified salmon gill poxvirus). However, we found no trace or remnant gene for A25/A26 in clade II viruses, indicating that their loss is an ancient modification occurring early in the radiation of these viruses. The absence of the entire A25/A26 differentiation system in clade II chordopoxviruses suggests a subsequent adaptation that permits transmission in the wild by the same EV morphotype that is responsible for intrahost dissemination. Interestingly, clade II viruses are known to be transmitted by biting insects, a mode of transmission in which virions are not exposed to the environment and where enhanced stability is not required. Obligate use of vectors may have rendered A26 functions redundant or even detrimental, leading to its exclusion. A26-mediated virus maturation therefore appears to be an evolutionary adaptation that explains poxvirus transmission modes and a signature for vector-adapted species.

Examination of the A25/A26/A27 locus shows that the phylogenetically clustered clade I molluscipoxviruses and avipoxviruses lack homologs of the OPXV A27 protein, while crocodylidpoxviruses possess a longer open reading frame (ORF) with weak homology (Fig. 6). Interestingly, the C-terminal domain of A27 shows similarity to the avipoxvirus and molluscipoxvirus A26 proteins (61), suggesting that A27 functions were initially embedded within A26 and segregated later after gene duplication. The fact that the A27 N-terminal domain (required for EV formation) is not conserved in these viruses suggests that either EVs are disrupted or are produced via an alternative mechanism. The transmission of crocodylidpoxviruses and MCV, the sole remaining poxvirus of humans, is poorly understood. However, MCV-infected cells become packed with single-membraned virus with no evidence of EV, suggesting it has evolved a highly specialized dissemination/transmission system that would benefit from molecular analysis. While our understanding of MCV can encompass disruption of EV, the implications for avipoxviruses, and the value of retaining F13 and A25/A26 having lost A27, are less clear and invite further investigation.

The function of ATI, providing a cytoplasmic inclusion in which MV is sequestered, is mirrored by entomopoxvirus spheroidins and baculovirus occlusion bodies (OBs). However, chordopoxvirus ATI and entomopoxvirus spheroidins are unrelated to each other (62) and may have arisen through convergent evolution. Entomopoxviruses and baculoviruses also produce two virion morphotypes, one responsible for intrahost dissemination and another sequestered inside OBs and responsible for host-to-host transmission (63–66). It is interesting to consider if entomopoxviruses and baculoviruses also code for a system regulating the balance and maturation of the two forms. However, in some entomopoxviruses, virions are occluded in OBs before they mature, and maturation continues after occlusion (66, 67). This suggests that in at least some entomopoxvirus species, occlusion is a passive process and there is no actively regulated differentiation of virion morphotypes. Interestingly, the A25 and A26 proteins are very closely related and are likely to have arisen as a duplication of an original single gene. Such an event would have facilitated the acquisition of differentiation functions. A26-mediated differentiation in chordopoxviruses may thus have arisen as an adaptation of a previously existing passive occlusion system.

## MATERIALS AND METHODS

### Cells and viruses.

BS-C-40, HEK293T, and RK13 cells were maintained in Dulbecco’s modified Eagle medium (DMEM, Life Technologies) supplemented with 10% heat-inactivated fetal bovine serum (FBS, Seralab), 100 U/ml penicillin, and 100 μg/ml streptomycin (Life Technologies). VACV strain Western Reserve (WR) and WR lacking F13 (a gift from Bernard Moss) have been described (21, 68). Viruses were propagated and titrated as PFU by plaque assay in monolayers of BS-C-40 cells. Infected cells were incubated for 48 to 72 h in DMEM containing 2.5% FBS and 1.5% carboxy-methyl cellulose (Sigma) and were stained with 5% crystal violet (Sigma).

### Plasmids for recombination.

All plasmids used to generate recombinant viruses were created in the background of a pUC13 transfer vector containing the Escherichia coli xanthine-guanine phosphoribosyltransferase (Ecogpt) gene fused with enhanced green fluorescent protein (EGFP) driven by the p7.5 promoter of VACV. To study F13 expression, transfer vectors encoding F13 fused with three copies of the FLAG epitope or the V5 epitope under the control of the natural F13 or A26 promoters were created as follows. The F13 left flank and coding sequence (nucleotides 40,834 to 42,325) within WR excluding the terminal STOP codon was amplified using Platinum HiFi *Taq* polymerase (Invitrogen) and oligonucleotides containing BamHI and NotI restriction sites. A piece of DNA containing three copies of the FLAG epitope followed by a STOP codon and a 276-bp sequence homologous to the right flank of F13 (40,555 to 40,830) flanked by NotI and XbaI was synthesized (GeneArt, Life Technologies) and cloned in frame with the F13 coding sequence to fuse the 3×FLAG tag to the C terminus of F13 via a 3×alanine linker. The 3×FLAG was excised using NotI and an internal SacII site and replaced for the V5 tag using overlapping oligonucleotides flanked with the same restriction sites. To introduce the A26 natural promoter (139,961 to 139,981) in lieu of the F13 promoter, the left flank of F13 excluding its promoter (41,966 to 42,325) and the F13 coding region from its ATG initiation codon until an internal BspEI site were amplified separately using oligonucleotides containing complementary strands of the A26 promoter. The PCR products were then annealed and reamplified, and the resulting product containing the A26 natural promoter in front of the F13 gene was cloned using the BamHI and BspEI sites. To study A26 expression, a DNA fragment containing the A26 left flank, including the A26 natural promoter (139,961 to 140,312) followed by an N-terminal 3×FLAG tag in frame with the A26 coding sequence via a 3×alanine linker containing a NotI site was designed and purchased from GeneArt and cloned into the pUC13 transfer vector. An identical fragment, with the exception that it contained the 21-bp F13 promoter including the ATG initiation codon (41,947 to 41,966) in lieu of the A26 natural promoter, was also purchased and cloned. All plasmids were verified by sequencing.

### Generation of recombinant viruses.

All recombinant viruses were generated by transient dominant selection (69). Ecogpt provided resistance to mycophenolic acid, hypoxanthine, and xanthine (Sigma), and EGFP allowed plaque visualization. Approximately 1 × 10^6^ HEK293T cells were infected with WR lacking *F13L* at a multiplicity of infection of 0.01 PFU per cell and were subsequently transfected with 10 μg of transfer vector containing F13 under cognate or heterologous promoters using LT1 (Mirus). The cells were incubated in DMEM supplemented with 2.5% FBS until cytopathic effect was detected. The progeny virus was used to infect BS-C-40 monolayers pretreated with Ecogpt-selective medium and incubated for 48 to 72 h in DMEM containing 2.5% FBS and 1.5% low-gelling temperature agarose (Sigma) until large fluorescent plaques were visualized using a Zeiss TV100 fluorescence microscope (Zeiss). Plaques were isolated and viruses subjected to at least three rounds of plaque purification to ensure stability. Purified plaques were then subjected to the same process in the absence of selective medium until large nonfluorescent plaques were identified, indicating that no foreign marker genes remained in the virus. These plaques were isolated and purified, and their identity was initially confirmed by amplification of the F13 locus and subsequent digestion exploiting the HpaI site in the F13 natural promoter and/or the NotI site introduced in the 3×alanine linker and finally by sequencing. Viruses were then expanded and titrated by plaque assay. To introduce A26 under cognate or heterologous promoters and to generate revertant viruses where natural promoters were reinstating, the same process was followed using the corresponding transfer vector and starting virus. When indicated, infected cell pellets were resuspended in ice-cold 10 mM Tris (pH 9.0) and homogenized with a Dounce tissue grinder. Clear lysates were purified through a 36% sucrose cushion and were resuspended in phosphate-buffered saline (PBS).

### Virus growth curves.

For the one-step growth curves, confluent BS-C-40 monolayers were infected in triplicate with 5 PFU per cell. At the indicated times postinfection, cells were scrapped in their own medium and collected by centrifugation. The supernatant was removed, and cell-associated virus was titrated by plaque assay on BS-C-40 cells after three rounds of freeze thawing. For the multistep growth curves, confluent BS-C-40 monolayers were infected in triplicate with 0.001 PFU per cell. At the indicated times postinfection, medium was collected and immediately titrated on BS-C-40 monolayers as extracellular virus. Cell-associated virus was titrated as above. Data are representative of at least two independent experiments each performed in triplicate.

### Plaque size assay.

BS-C-40 monolayers were infected with 50 PFU of virus per well for 72 h to allow formation of individual plaques. The cells were then stained with crystal violet, and the stained monolayers were placed in a flat-bed scanner and scanned at 1,200 dots per inch (dpi). Images were analyzed in ImageJ software, and the scale was calibrated for a 1-mm area (approximately 47 pixels per mm). Plaque area was measured using the freehand selection tool, and the resulting measurement data were imported into GraphPad for further analysis. Data are representative of at least three independent experiments and were statistically analyzed by Student’s *t* test.

### Measurement of actin tails.

RK-13 cells were infected with 5 PFU per cell of wild-type or vA26^pF13^ and incubated for 24 h at 37°C. The cells were then fixed with 4% paraformaldehyde and stained with Phalloidin-iFluor 488 (Invitrogen). To enumerate the actin tails produced, a total of 70 cells were imaged per virus. The number of actin tails within each field of view were counted and plotted.

### Kinetics of expression by immunoblotting.

Confluent BS-C-40 monolayers were prechilled for 60 min and infected with 5 PFU per cell of virus at 4°C. The cells were incubated for 60 min at 4°C and then shifted to 37°C for a further 60 min to initiate infection synchronously. The inoculum was then aspirated and replaced with DMEM containing 2.5% FBS, and cells were collected at the indicated time points postinfection and subjected to SDS-PAGE. Cells were lysed in 50 mM Tris-HCl (pH 7.5), 150 mM NaCl, and 1% Triton X-100 (vol/vol) supplemented with protease inhibitors (Roche). Cleared lysates were mixed with Laemmli buffer, denatured, and analyzed by SDS-PAGE as previously described (70). To detect the A26-A27 interaction, lysis buffer was further supplemented with 100 mM *N*-ethylmaleimide (Sigma) as previously described (35), and cleared lysates were mixed with lithium dodecyl sulfate (LDS) sample buffer (Invitrogen) and kept undenatured. The following primary antibodies were used: anti-F13 has been described previously (30), anti-A27 monoclonal antibody was made in-house by D.U., anti-C6 was a gift from Geoffrey Smith (71); anti-FLAG (Sigma; 1:20,000), anti-V5 (Sigma; 1:5,000), and anti-β-tubulin (Abcam; 1:5,000). Primary antibodies were detected using IRDye-conjugated secondary antibodies in an Odyssey infrared imager (Li-COR Biosciences). Quantitative data from the images were obtained using Odyssey software.

### Kinetics of expression by real-time PCR.

Confluent BS-C-40 monolayers were prechilled for 60 min and infected with 2 PFU per cell of virus at 4°C. The cells were incubated for 60 min at 4°C and then shifted to 37°C for a further 60 min to initiate infection synchronously. The inoculum was then aspirated and replaced with DMEM containing 2.5% FBS. The cells were collected at the indicated time points postinfection, and cDNA was produced as previously described (72). cDNA was diluted 5-fold in water and used as a template for PCR using GoTaq DNA polymerase (Promega) and specific primers for the indicated viral genes and *GAPDH*.

### Statistical analysis.

Representative results from at least two independent experiments are shown unless otherwise indicated in the figure legends. Data were analyzed using GraphPad Prism software, and an unpaired Student’s *t* test was used to determine significance between groups.

### Computational analysis.

For E9, A3, and A10 phylogenetic analysis, viral sequences were downloaded from NCBI, and suitable representatives for each virus/host species were selected, as shown in Table 1. We also downloaded from Virus Pathogen Database and Analysis Resource (ViPR; https://www.viprbrc.org/) ortholog groups for vaccinia proteins E9, A3, A10, F13, A24, A25, A26, A27, and A28. Each of them was turned into a hidden Markov model (HMM) protein model by using HMMER (73) version 3.1b2. In order to identify homologs to vaccinia virus proteins, we first listed all ORFs for each viral genome from all complete poxvirus genome sequences in the ViPR database and then translated them into protein sequence. We then conducted HMMER searches, locating hits for each HMM protein model in all viral genomes. The hits were filtered for length and quality. As proteins A25, A26, and A27 show a high degree of paralogy due to the presence of common subdomains, to produce Fig. 7, whenever ambiguities arose we selected the attribution that maximized HMMER score and length similarity. The phylogenetic trees for proteins E9, A3, and A10 were produced by first computing multiple alignments with Clustal Omega version 1.2.3 (74) for the sequences of each protein and then building an (approximate) maximum likelihood (ML) tree based on the JTT model of amino acid evolution with FastTree version 2.1.9 (75). The plots were generated with ggtree (76).

**TABLE 1:** Poxvirus species (name, accession number, and genome length) used for phylogenetic analyses

Species	Accession	Length	Species	Accession	Length
Akhmeta	MH607143	217,740	Ectromelia	NC_004105	209,771
Cowpox	NC_003663	224,499	Variola	X69198	185,578
Taterapox	DQ437594	198,050	Camelpox	AF438165	205,719
Monkeypox	AY603973	199,195	Vaccinia	AY243312	194,711
Buffalopox	MG599038	195,630	Skunkpox	KU749310	222,832
Raccoonpox	KP143769	214,699	Volepox	KU749311	217,124
Yoka pox	HQ849551	175,699	NY pox	MF001305	200,223
Yaba monkey tumor	AY386371	134,721	Tanapox	EF420156	144,565
Murmansk pox	MF001304	204,055	Rabbit fibroma	AF170722	159,857
Myxoma	AF170726	161,773	Swinepox	AF410153	146,454
Deerpox	AY689436	166,259	White-tailed deer pox	MF966153	163,340
Moosepox	MG751778	164,258	Goatpox	AY077835	149,599
Sheeppox	NC_004002	149,955	Lumpy skin disease	AF409137	150,793
BeAn	KY094066	163,005	Sea otter pox	MH427217	127,879
Cotia	KM595078	185,139	Pteropox	KU980965	133,492
Bovine popular stomatitis	AY386265	134,431	Seal parapox	KY382358	127,941
Red deer parapox	KM502564	139,981	Pseudocowpox	NC_013804	145,289
Orf	AY386264	139,962	Western grey kangaroo pox	MF467280	170,141
Eastern grey kangaroo pox	MF661791	170,681	Turkeypox	KP728110	188,534
Shearwaterpox	KX857216	326,929	Penguinpox	KJ859677	306,862
Canarypox	AY318871	359,853	Pigeonpox	KJ801920	282,356
Fowlpox	AF198100	288,539	Flamingopox	MF678796	293,123
Saltwater crocodile pox 1	MG450915	187,976	Saltwater crocodile pox 2	MG450916	184,894
Nile crocodile pox	DQ356948	190,054	Anomala cuprea entomopox	AP013055	245,717
Mythimna separata entomopox	HF679134	281,182	Adoxophyes honmai entomopox	HF679131	228,750
Amsacta moorei entomopox	AF250284	232,392	Choristoneura biennis entomopox	HF679132	307,691
Choristoneura rosaceana entomopox	HF679133	282,895	Salmon gill pox	KT159937	241,564
Molluscum contagiosum 1	NC_001731	190,289	Molluscum contagiosum 2	MH320549	193,271
